# Frequency and Spectrum of KRAS Mutations in Moroccan Patients with Lung Adenocarcinoma

**DOI:** 10.1155/2014/192493

**Published:** 2014-03-05

**Authors:** Ibrahim Elghissassi, Hanane Inrhaoun, Anwar Boukir, Fouad Kettani, Lamia Gamra, Amina Mestari, Lamia Jabri, Youssef Bensouda, Hind Mrabti, Hassan Errihani

**Affiliations:** ^1^Medical Oncology Department, National Institute of Oncology, Allal Elfassi Street, 62000 Rabat, Morocco; ^2^Pathology Department, Nations-Unies Pathology Center, Rabat, Morocco; ^3^Pathology Department, Hassan Pathology Center, Rabat, Morocco; ^4^Pathology Department, Agdal Pathology Center, Rabat, Morocco; ^5^Pathology Department, Casapath Pathology Center, Casablanca, Morocco

## Abstract

*Background*. In lung adenocarcinoma, the frequency of KRAS mutations is ethnicity dependent with a higher proportion in African Americans and white Caucasians than in Asians. The prevalence of these mutations among North Africans patients is unknown. The objective of this study was to report the frequency and spectrum of KRAS mutations in a group of Moroccan lung adenocarcinoma patients. *Methods*. Tumor specimens from 117 Moroccan patients with lung adenocarcinoma were selected to determine frequency and spectrum of KRAS mutations. KRAS mutations in codons 12 and 13 of exon 2 were analyzed using conventional DNA sequencing. *Results*. The overall frequency of the KRAS mutations was 9% (11/117). In the population with KRAS mutations, there was a trend towards more male (*P* = 0.06) and more smokers (*P* = 0.08) compared to patients with wild type KRAS. KRAS mutations were located at codon 12 in 10 out of 11 patients (91%). The G12C mutation was the most frequent KRAS mutation (73%). *Conclusion*. This is the first study to date examining the frequency and spectrum of KRAS mutations in lung adenocarcinomas in North African and Arab populations. KRAS mutation frequency in Moroccan patients was comparable with the frequency observed in East-Asian population. KRAS mutations are more likely observed in males and smokers and to be transversions. Further studies, in larger numbers of patients, are needed to confirm these findings.

## 1. Introduction

Lung cancer is one of the most common cancers in Morocco and in the world and is the leading cause of cancer mortality in both males and females [1, 2]. During the last few years, improvement in the knowledge of lung cancer molecular biology led to identification of several biological events crucial for tumor cell survival.

In nonsmall-cell lung cancer (NSCLC), one of the most commonly genetic aberrations is conversion of the protooncogene KRAS to its activated oncogenic form. KRAS encodes low molecular weight GTPase binding proteins that regulate cell growth, differentiation, and apoptosis. Mutations of KRAS are associated with impaired GTPase activity, causing increased mitogenic RAS signaling [3].

Mutations in KRAS gene occur more frequently (20–30%) in adenocarcinoma and less frequently (about 7%) in squamous-cell carcinoma and are usually associated with a history of smoking [4]. The incidence of KRAS mutation is ethnicity dependent with a higher proportion in African Americans and white Caucasians (20–30%) than in Asians (5–20%) [5–7]. However, such information is still lacking in some other races, such as North African patients.

Typically, KRAS mutations are a point mutation in the gene replacing an amino acid at positions 12, 13, or 61 [8]. Somatic KRAS mutations predominantly involve codons 12 and 13. Together, these two mutations account for nearly 95% of all KRAS mutations in lung cancer. The G12C mutation in codon 12 is by far the most frequent mutation in NSCLC [8–10].

The main goal of this study was to report the frequency and spectrum of KRAS mutations in unselected group of Moroccan lung adenocarcinoma patients. In addition, we have examined the relationship between the mutations and some parameters, including gender and smoking status.

## 2. Methods

### 2.1. Patients and Tumor Samples

Data of all lung adenocarcinoma samples were collected from four pathology laboratories in Morocco between November 2010 and August 2012. The participating laboratories were Nations-Unies Pathology Center, Rabat; Hassan Pathology Center, Rabat; Agdal Pathology Center, Rabat; Casapath Pathology Center, Casablanca.

All of these laboratories outsource KRAS mutation testing (Molecular Biology Laboratory in Paris, France) and perform more than 90% of all tests requested in Morocco.

Only Moroccan metastatic lung adenocarcinoma patients were included. The histological diagnosis of adenocarcinoma was verified using the pathology report provided by participating laboratories.

Never smokers were defined as individuals who reported smoking fewer than 100 cigarettes in their lifetime.

### 2.2. KRAS Mutation Analysis

Genomic DNA was extracted from tumors tissues according to standard procedures. KRAS mutations in codons 12 and 13 of exon 2 were analyzed using conventional DNA sequencing.

### 2.3. Ethics Statement

The ethics committee approval was not necessary for this study, since all mutation tests were part of the routine diagnostic procedure and all patient and tumor characteristics were collected anonymously.

### 2.4. Statistical Analysis

The potential associations of KRAS mutation with age, gender, and smoking status were analyzed using Fisher's exact test. Means of age were compared using the *T* test. A *P* value less than 0.05 was considered statistically significant. All analyses were performed using SPSS Version 20.

## 3. Results

One hundred and seventeen lung adenocarcinomas from Moroccan patients were collected. The study population consisted of 77 men (66%) and 40 women (34%), with a median age of 59 years (range: 37 to 87 years). The demographic characteristics of the patients are provided in Table 1.

A total of 11 KRAS mutations were found in this series of 117 patients (9%). KRAS mutations were identified in 13% of smokers (9/68) and 4% of never smokers (2/49). There were 13% of male (10/77) and 2% of female (1/40) with KRAS mutations.

In the population with KRAS mutations, there was a trend towards more male than female compared to patients with wild type KRAS (91% versus 63%, *P* = 0.06) but this did not reach statistical significance. The frequency of smokers in patients with tumors having KRAS mutations was higher than that observed in patients without mutations (82% versus 56%, *P* = 0.08) but also this did not reach statistical significance. The mean age did not differ between the two groups (Table 2).

All KRAS mutations were located at codon 12, with the exception of a single patient in which KRAS was mutated at codon 13. The G12C mutation was the most frequent KRAS mutation (73%) in this series (Table 3).

## 4. Discussion

In the present series, KRAS mutations were identified in 11 of 117 Moroccan patients (9%). To the best of our knowledge, this study represents the first study to date reporting KRAS mutation rate in North African and Arabs populations.

Some previous studies have demonstrated a genetic divergence of KRAS mutation rates according to ethnicity [11–18]. Indeed, KRAS mutations rate in Caucasian (26–37%) and African-American (17–37%) patients is much higher than that in East-Asian population (6–11%). KRAS mutation frequency in Moroccan patients, as reported in this study, was comparable with the frequency observed in East-Asian population (Table 4).

In the population with KRAS mutations, we found a trend towards more male than female compared to patients with wild type KRAS (91% versus 63%, *P* = 0.06). This did not reach statistical significance, probably due to the low number of patients with KRAS mutations in the present study. However, these results are in keeping with some published reports showing a trend towards more mutations in males [19, 20].

In this study, the higher frequency of KRAS mutations among smokers (82%) supports data from some previous studies. For example, Ahrendt et al. reported a strong positive association between presence of KRAS mutations and smoking [21]. A meta-analysis of KRAS mutations in NSCLC reported a frequency of 26% in tumors of current/former smokers and 6% in tumors of never smokers [22]. On the other hand, KRAS mutations have been detected in a significant proportion of never smoker lung adenocarcinoma patients, with an incidence up to 15% [7], and thus remain not rare in this subpopulation.

In lung adenocarcinomas, the majority of KRAS mutations have been identified in codons 12 and 13. In our subjects, the distribution of mutations of KRAS at codons 12 (10/11 (91%)) and 13 (1/11 (9%)) appear to be comparable with the frequency of the other studies [23, 24]. Furthermore, the G12C mutation was the most frequent KRAS mutation (73%) confirming previous results [8–10].

Transversions (substituting a pyrimidine base for a purine base or vice versa) are more common than transitions (substituting purine for purine or pyrimidine for pyrimidine). KRAS mutations observed in smokers are more likely to be transversions unlike the transitions more common in patients with no history of cigarette smoking [7]. Indeed, the etiology of G → T transversion in lung cancer has been attributed to the exposure to polycyclic aromatic hydrocarbons (PAH) present in tobacco smoke [25]. In the present study, 89% of smokers (8/9) had a transversion mutation supporting these data.

Till date, the clinical applicability of the KRAS mutation is limited. Firstly, there is no specific treatment currently available for NSCLC with KRAS mutations. Secondly, the prognostic and predictive role of KRAS mutations in NSCLC remains controversial. While some studies suggested that mutant KRAS may have a potential negative prognostic effect and could be predictive of lack of response to chemotherapy and epidermal growth factor receptor (EGFR) inhibitors, other studies failed to confirm this result [6, 26]. However, previous studies have shown that EGFR and KRAS mutations are mutually exclusive [5, 19]. KRAS may therefore have a role as part of an efficient testing algorithm.

In Summary, the present study revealed that KRAS mutation frequency in Moroccan patients was comparable with the frequency observed in East-Asian population. In keeping with the literature, KRAS mutations are more likely observed in males and smokers and to be transversions. Further studies, in larger numbers of patients, are needed to confirm these findings.

## Figures and Tables

**Table 1:** Demographic characteristics of the study population.

	All patients (*n* = 117)	
	No	%
Age (years)		
Median	59	
Range	37–87	
Sex		
Male	77	(66%)
Female	40	(34%)
Smoking history		
Never	49	(42%)
Ever	68	(58%)
Histology		
Adenocarcinoma	117	(100%)
Stage at diagnosis		
** **IV	117	(100%)

**Table 2:** Patient characteristics by KRAS mutation status.

	KRAS mutations (*n* = 11)		Wild type (*n* = 106)		*P *
	No	%	No	%	
Mean age at diagnosis	60		60		*P* = 0.98*
Sex					
Male	10	(91%)	67	(63%)	*P* = 0.06**
Female	1	(9%)	39	(37%)	
Smoking history					
Ever	9	(82%)	59	(56%)	*P* = 0.08**
Never	2	(18%)	47	(44%)	

*P value calculated by *t*-test.

**P value calculated by Fisher's exact test.

**Table 3:** Frequency of KRAS mutation type.

KRAS		Smokers	Never smokers	Total (%)
Mutation	Nucleotide			
Codon 12				
G12C	**G**GT → **T**GT	8	0	8 (73%)
G12S	**G**GT → **A**GT	1	0	1 (9%)
G12D	G**G**T → G**A**T	0	1	1 (9%)
Codon 13				
G13D	G**G**C → G**A**C	0	1	1 (9%)

**Table 4:** The frequency of KRAS mutations by race.

Study	Race	KRAS Mutation (%)
Smits et al. [11]	Caucasian	244/661 (37%)
Boch et al. [12]	Caucasian	67/254 (26%)
Reinersman et al. [13]	Caucasian	125/476 (26%)
Hunt et al. [14]	African-American	22/60 (37%)
Leidner et al. [15]	African-American	12/53 (23%)
Reinersman et al. [13]	African-American	21/121 (17%)
Sasaki et al. [16]	East Asian	21/190 (11%)
Kim et al. [17]	East Asian	5/71 (7%)
Akamatsu et al. [18]	East Asian	2/31 (6%)
This study	North African	11/117 (9%)
